# Genital invasion or perigenital spread may pose a risk of marginal misses for Intensity Modulated Radiotherapy (IMRT) in anal cancer

**DOI:** 10.1186/s13014-016-0628-4

**Published:** 2016-04-04

**Authors:** Julia Koeck, Frank Lohr, Daniel Buergy, Karen Büsing, Marcus J. Trunk, Frederik Wenz, Sabine Mai

**Affiliations:** Department of Radiation Oncology, University Medical Center Mannheim, University of Heidelberg, Theodor-Kutzer-Ufer 1-3, 68135 Mannheim, Germany; Department of Clinical Radiology and Nuclear Medicine, University Medical Center Mannheim, University of Heidelberg, Mannheim, Germany; Institute of Pathology, University Medical Center Mannheim, University of Heidelberg, Mannheim, Germany

**Keywords:** Anal carcinoma, Intensity Modulated Radiotherapy (IMRT), Genital sparing, Vulvar relapse, Perigenital spread, Lymphatic spread, Marginal miss

## Abstract

**Background:**

While intensity modulated radiotherapy (IMRT) in anal cancer is feasible and improves high-dose conformality, the current RTOG/AGITG contouring atlas and planning guidelines lack specific instructions on how to proceed with external genitalia. Meanwhile, the RTOG-Protocol 0529 explicitly recommends genital sparing on the basis of specific genital dose constraints. Recent pattern-of-relapse studies based on conventional techniques suggest that marginal miss might be a potential consequence of genital sparing. Our goal is to outline the potential scope and increase the awareness for this clinical issue.

**Methods:**

We present and discuss four patients with perigenital spread in anal cancer in both early and advanced stages (three at time of first diagnosis and one in form of relapse). Genital/perigenital spread was observed once as direct genital infiltration and thrice in form of perigenital lymphatic spread.

**Results:**

We review the available data regarding the potential consequences of genital sparing in anal cancer. Pattern-of-relapse studies in anal cancer after conventional radiotherapy and the current use of IMRT in anal cancer are equivocal but suggest that genital sparing may occasionally result in marginal miss. An obvious hypothesis suggested by our report is that perigenital lymphovascular invasion might be associated with manifest inguinal N+ disease.

**Conclusions:**

Local failure has low salvage rates in recent anal cancer treatment series. Perigenital spread may pose a risk of marginal misses in IMRT in anal cancer. To prevent marginal misses, meticulous pattern-of-relapse analyses of controlled IMRT-series are warranted. Until their publication, genital sparing should be applied with caution, PET/CT should be used when possible and meeting genital dose constraints should not be prioritized over CTV coverage, especially (but not only) in stage T3/4 and N+ disease.

## Background

Organ preserving combined modality treatment has proven to be as effective as radical surgery and has been introduced as standard therapy for anal cancer worldwide [1–4]. Anal cancer is currently a highly curable neoplasm with radiotherapy and concurrent chemotherapy with 5-fluorouracil (5FU) and mitomycin C (MMC). Disease free survival lies between 70 and 90 %, depending on tumor size [5–8]. In modern series, salvage rates for local relapse are lower than in historic series, which probably is a consequence of better overall results based on changing biology with increasing infections with human papillomavirus (HPV) and improved treatment quality, resulting in less but more therapy-resistant relapses. Therefore, local relapse dramatically influences overall survival [9].

When compared to standard anterior-posterior/posterior-anterior (ap-pa) or 3D-conformal radiotherapy (3D-CRT, typically with a 3-field or 4-field box technique), intensity modulated radiotherapy (IMRT) provides similar coverage of the planning target volume (PTV) while better sparing organs at risk (OAR), reducing the dose to critical structures [10–19] and according to an initial report of the RTOG (Radiation Therapy Oncology Group) phase II trial 0529 (though not meeting the primary endpoint) reducing acute toxicity as a consequence [20]. As IMRT can produce a highly conformal dose distribution with steep dose gradients outside the target volume there is the need for meticulously defining PTV and OAR. RTOG-0529 also demonstrated the complexity of the whole treatment planning chain with 81 % of plans that had to be modified after initial review [20]. Marginal misses as a consequence of improved dose conformality have already been observed in other tumor entities such as head and neck cancer where IMRT is now the gold standard [21, 22], and slightly inferior tumor control rates with IMRT in some though not all randomized trials might be a result of a change in irradiated volumes [23–25].

The Australasian Gastrointestinal Trials Group (AGITG) and the RTOG have recently established contouring-guidelines for IMRT in anal cancer [26, 27]. The protocol of RTOG-0529 lists several dose constraints for critical normal structures including the external genitalia and requests that in every patient an effort should be made to achieve them [20].

Anal cancer invading genitalia or spreading into lymphatic structures close to external genitalia has been observed several times at our department over the last 18 months. All patients had extensive clinical and imaging workup including FDG-PET/CT (positron emission tomography/computed tomography) and MRI (magnetic resonance imaging), for the first time providing the opportunity to obtain detailed objective visualization of genital/perigenital spread [28]. As local relapse dramatically influences overall survival, as recently shown by Mai et al. [9], avoiding marginal misses becomes a crucial factor in treatment planning in IMRT of anal cancer. To outline the potential scope and increase the awareness for this clinical issue, in this report we present one case of anal carcinoma with a perivulvar/vulvar relapse *after* IMRT and three cases of anal cancer with (peri)genital infiltration at the time of first diagnosis *before* IMRT treatment and discuss them in the context of recently published relevant data. All patients have consented with their regular informed consent to anonymous scientific analysis of their data.

## Case reports

### Direct genital infiltration

#### Case 1

**Fig. 1:**
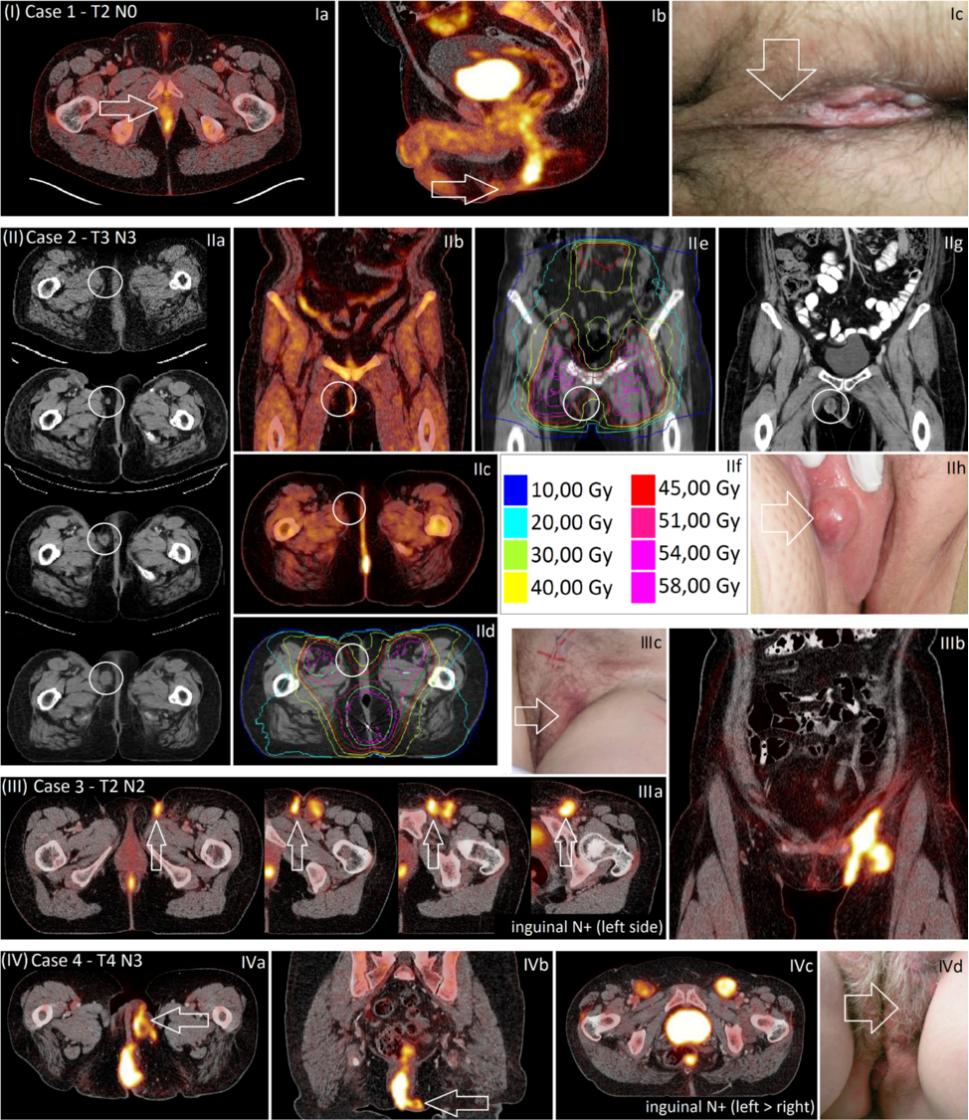
Direct genital infiltration and perigenital involvement and/or perivulvar relapse in anal carcinoma: PET/CT images and clinical manifestation. Legend: (I) Direct genital infiltration in N0/N3 anal cancer at time of first diagnosis (case 1) with PET/CT images in transversal and sagittal planes (Ia, b) and clinical photo (Ic); (II) Vulvar/perivulvar relapse 9 months after concurrent IMRT and chemotherapy of an inguinally N+ anal cancer (case 2) with the CT-sequence of the development of the relapse from time of first diagnosis until 9 months after radiochemotherapy (IIa), pretherapeutic PET/CT images in transversal and coronal planes (IIb, c), IMRT plans in transversal and coronal planes with isodose details (IId, e, f), CT image of the relapse in coronal plane and clinical photo at 9 months after therapy (IIg, h); (III) and (IV) Perigenital involvement related to lymphatic spread in inguinally N+ anal cancer at time of first diagnosis (case 3 and case 4) with PET/CT images in transversal and coronal planes (IIIa, b and IVa, b, c) and clinical photo (IIIc and IVd)

A 50-year-old male patient presented with T2N0M0 anal margin squamous cell carcinoma (SCC) in 04/2013, with the tumor reaching the posterior scrotal skin (Fig. 1, Ia-c). He received concurrent radiochemotherapy with 2 cycles of 5FU/MMC and IMRT with 36 Gy to the pelvis and inguinal lymph nodes, followed by 45 Gy to the primary tumor and pelvis (without inguinal lymph nodes) and a perineal electron boost to the macroscopic tumor up to 50.4 Gy. The area of the scrotal skin that was considered infiltrated was included in the boost field. Follow-up has been without signs of relapse.

### Perigenital involvement related to lymphatic spread

#### Case 2

A 52-year-old female patient presented with T3N3M0 anal canal SCC in 03/2012. She received IMRT with 45 Gy to the pelvis (including inguinal and paraaortic lymph nodes) and a boost up to 54 Gy to the primary tumor and PET-positive lymph nodes. Two concurrent and two sequential cycles of 5FU/MMC chemotherapy were administered. The second follow-up at four months with FDG-PET/CT showed an elevated SUV in most of the initially affected lymph nodes and a biopsy of the anal canal revealed persisting carcinoma. Salvage surgery was performed (abdominoperineal resection and systematic inguinal lymphadenectomy). Due to a postoperatively persisting lymphatic fistula in the left inguinal region, a course of obliterating radiotherapy was planned. The planning CT unexpectedly showed a small subcutaneous tumor in the right vulvar/perivulvar region. A control CT 4 weeks after radiotherapy showed a significant increase in size, suggesting locoregional relapse (Fig. 1, IIa-h). As surgery was not considered an option due to elevated risk of wound healing impairment, she received low-dose-rate brachytherapy (10 Gy). Local control and improvement of local wound situation were achieved for several weeks. As she developed multilocular failure some weeks later, palliative chemotherapy was initiated. A few months later, she died in palliative care.

#### Case 3

**Fig. 2:**
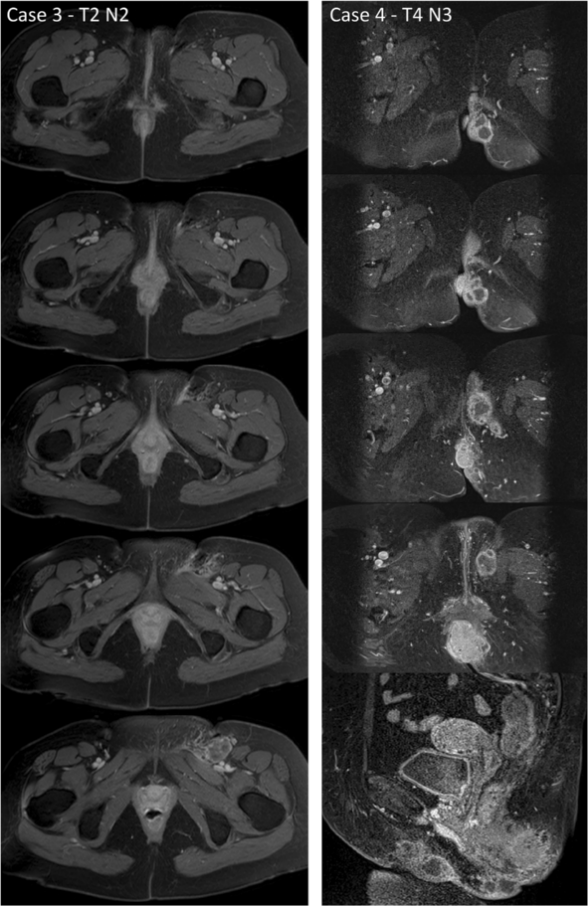
Perigenital involvement in anal carcinoma: MR images Legend: Representative MR images of case 3 (*left*) and case 4 (*right*) in transversal and sagittal planes showing perigenital involvement in anal carcinoma in a T1 weighted sequence after administration of contrast agent

A 55-year-old female patient presented with a T2N2M0 anal canal SCC in 09/2012. A PET/CT showed an affected inguinal lymph node and perigenital lymphatic infiltration on the left side (Fig. 1, IIIa, b). Fig. 2 shows MRI images of the perigenital infiltration (Fig. 2, left side). Clinically, the patient also had tumor infiltration of the left mons pubis (Fig. 1, IIIc). IMRT was applied with 45 Gy to the pelvis and both inguinal lymphatic regions, with a boost up to 54 Gy to the PET-positive primary tumor and left inguinal lymph nodes. Two concurrent and two sequential cycles of chemotherapy with 5FU/MMC were administered. Follow-up has been without signs of relapse.

#### Case 4

In 02/2013, a 69-year-old female patient presented with T4N3M0 anal canal SCC with infiltration of the vagina and PET-positive inguinal lymph nodes with left perivulvar infiltration (Fig. 1, IVa-d). Fig. 2 shows MRI images of the perigenital infiltration (Fig. 2, right side). IMRT was applied to the pelvis including the inguinal lymph nodes on both sides up to 45 Gy and a boost to the primary tumor including the vulva and all PET-positive lymph nodes up to 54 Gy. As one concurrent 5FU/MMC chemotherapy cycle lead to febrile leucopenia, urosepsis and cardiac complications, chemotherapy was concluded at 3 cycles of cisplatin (week 7, 14, 19). After 3 months the patient presented with progressive PET-positive paraaortic and presacral lymph nodes and at 4 months a residual tumor in the anal canal was diagnosed. Additionally, clinical examination showed new small vulvar nodes, which were biopsied and classified as vulvar intraepithelial neoplasia (VIN). The patient received salvage surgery (abdominoperineal resection and removal of the large left labium). Upon follow-up at six weeks after salvage surgery, the patient presented with progressive inguinal lymph nodes. A solitary pulmonary metastasis was resected in 11/2013. Due to further progressive disease in 01/2014, she received palliative chemotherapy and died several months later.

## Discussion

Evidently, the four cases reported here cannot be representative for the whole group of patients with genital involvement in anal cancer, but are certainly hypothesis-generating. They clearly demonstrate that involvement of external genitalia does in fact occur clinically, may be subtle, is not necessarily related to a large primary tumor and has therefore to be considered when defining target volumes.

Anal cancer spreads locoregionally both contiguously and along lymphatic vessels. Inguinal and pelvic nodes are at risk, especially for large primary tumors [29–32]. External genitalia can be involved for three potential reasons: synchronous genital primaries because of virally induced field cancerogenesis, direct invasion of the primary tumor and finally perigenital lymphatic spread that invades the genitals. In a study of Fenger et al., half of the female patients with anal intraepithelial neoplasia (AIN) had accompanying or previous neoplasia of the vulva or perineum [33], likely manifesting field cancerization as a consequence of HPV infection. Case 4 in our report may represent such a situation with anal cancer instead of AIN, as the HPV analysis (of both primary anal tumor and VIN) showed a HPV+ and p16+ tumor. However, as HPV is positive in about 90 % of the patients with anal cancer [34–36], a positive result for HPV in anal cancer doesn’t prove a direct link between the anal cancer and the genital disease. The incidence of synchronous HPV related tumors is not yet known and the other two reasons for genital invasion (direct infiltration and perigenital lymphatic spread) are likely more relevant for target delineation and shall therefore be discussed below more in detail.

In regard to diagnostic imaging for treatment planning in anal cancer, recommendations slightly differ in various international guidelines. The NCCN (National Comprehensive Cancer Network) guidelines recommend CT or MRI of the pelvis for evaluation of pelvic lymph nodes [26]. A FDG-PET/CT can assess N+ disease and also provides detailed visualization of genital/perigenital spread [32, 37]. The NCCN guidelines, updated in 2012 after a NCCN Anal Carcinoma Panel meeting, state after thorough interdisciplinary discussion that “PET/CT should be considered for treatment planning” [38, 39]. Mai et al. showed the possibility of dose reduction in inguinal lymph node regions on the basis of FDG-PET/CT [28]. In our opinion, performing a PET/CT for treatment planning therefore should be considered, when available.

As for patient setup, radiation treatment in anal cancer can be performed in prone or supine position with both advantages and disadvantages in each position [32]. Regarding genital sparing, a “frog legged” supine position allows avoiding unnecessary radiation dermatitis by separation of the medial thighs. For female patients, the use of a genital dilator may further improve vaginal sparing [40].

In the last decade, IMRT has become a widely used radiotherapy technique for various tumor entities such as head and neck, prostate or breast cancer [41]. The steep dose gradients created by IMRT bear the potential to increase the rate of marginal misses, and both anecdotal evidence [21, 22] and results from randomized trials [23, 24] have raised the awareness towards this issue. By now, IMRT is increasingly used for anal cancer within the framework of radiochemotherapy, both as step-and-shoot/dynamic IMRT and as volumetric modulated arc therapy (VMAT) [42]. Performing highly conformal radiotherapy with IMRT in anal cancer requires detailed knowledge of target structures for delineating the complex elective nodal regions without omitting any important tumor volume. There have recently been efforts within the RTOG and the AGITG to address the question as how exactly to contour the region of the primary tumor and elective target volumes while sparing femoral head and neck, bladder, bowel and external genitalia [26, 27]. In a contouring atlas, detailed recommendations and guidelines for delineating the PTV are given. Regarding OAR however, statements have so far remained vague. Up to now, there exist no specific data on how to best spare sensitive structures without compromising the target coverage. Only the protocol of RTOG-0529 lists several dose constraints for critical normal structures including the external genitalia and requests that in every patient an effort should be made to achieve them, without giving contouring recommendations: no more than 50 % of the external genitalia should receive a dose above 20 Gy, no more than 35 % a dose above 30 Gy, and no more than 5 % a dose above 40 Gy [20].

Although the primary endpoint of reducing grade 2 toxicity was not met, the initial data published for this RTOG-0529 multicenter trial suggest reduced grade 3 toxicity (likely even more important than the primary endpoint) with IMRT [20] when compared to the seminal RTOG-9811 trial where the conventional radiation treatment still was ap-pa or a 3D-conformal multifield technique [6, 43]. There have been various other clinical studies that indicate similar or reduced acute toxicities for IMRT when compared to 3D-CRT [11–15, 17, 18, 20, 44]. Finally, in a dosimetric study, Chen et al. explicitly showed that external genitalia can be spared by IMRT [10].

To better understand to what extent genitals can actually be spared from treatment, pattern-of-relapse analyses have to be performed. Two recent studies have reported such patterns-of-relapse in anal cancer after conventional, simulator based therapy. Relapses have mostly occurred locally in the area of the primary tumor or regionally in initially affected lymph node areas [45, 46] and can therefore most likely be considered in-field, which is to be expected given the large volumes treated with conventional techniques. Das et al. report that 75 % of relapses involved the anus or rectum, with or without involvement of other structures, without elaborating, though, on how many relapses might have been due to marginal misses. Their number is likely small, however, because the majority of dose was applied ap-pa and only part of the treatment was given with a 3-field technique with some anterior genital sparing [45]. Wright et al., on the other hand, apparently performed explicit genital blocking for a large part of the treatment. Locoregional failure overall was significantly associated with T-stage but not N-stage. In detail, patients with *external* perianal failure had stages from T1N0 up to T3N0 and the two patients with vulvar and scrotal relapse had T2N2 and T2N3 stage, respectively. Both patients with genital relapses had also both inguinal relapse and finally metastatic disease. Though an ultimately precise analysis of in-field relapse vs. marginal miss was not possible in their series either due to methodical limits (no rigorous image review, physician assessment only), they explicitly suggest that “three of five failures appear to be in-field and two marginal, primarily because of inadequate coverage anteriorly. Failures occurred up to 3 cm inferior to the anal verge and anteriorly into the scrotum or vulva. This highlights the need to respect a minimum of a 2-cm margin on the tumor and anal margin in the CTV, even if this makes meeting genital dose constraints difficult” [46].

Regarding IMRT, there is a body of retrospective data that reports local and regional control after IMRT for anal cancer, albeit still with relatively short follow-up, with a varying degree of genital sparing, a varying percentage of local and regional relapse and no explicit analysis to what extent genital sparing may have contributed to local and regional relapse [11–16, 19, 47–49]. A full publication of the post-IMRT tumor control data from RTOG-0529 cannot be found so far.

In the series presented here, one patient (case 2, initially T3N3M0) showed a vulvar/perivulvar *relapse* indicating marginal miss (Fig. 1, IIa, g, h). The relapse occurred thirteen months after primary diagnosis. The area of the relapse had been in the margin of the radiation field and had received approximately 41–46 Gy (Fig. 1, IId, f). As there had been no sign of vulvar/perivulvar tumor at time of first diagnosis (Fig. 1, IIb, c), nor at posttreatment up to 5 months, we assume microscopic perigenital lymphovascular invasion and vulvar relapse due to insufficient radiation dose. However, as the patient also had persisting tumor in the anal canal (high dose region), treatment failure in this case was likely caused by multiple tumor-biological factors and not only by marginal miss.

The other three cases report genital/perigenital infiltration already at time of first diagnosis, *before* application of IMRT, therefore treatment volumes could be chosen appropriately. In case 1 (T2N0M0), scrotal infiltration could not be ruled out clinically and consequently the clinically infiltrated parts of the scrotum/scrotal skin were included into the CTV. In case 3 (T2N2M0 with infiltration of the left inguinal lymph node), clinically there was considerable infiltration of the left mons pubis, demonstrating a case of perigenital lymphovascular invasion. The *right* inguinal region had shown no sign of affected lymph nodes at the start of the treatment but the patient was considered to be at higher risk of having further (microscopic) lymph node infiltration or inguinal relapse. Therefore, radiation on the right side purposely was performed not only with 36 Gy but up to 45 Gy, in order to apply a sufficient radiation dose to both inguinal regions. Both patients have been in remission to date, likely as a consequence of the relatively large treatment volumes and target contouring being based both on clinical and PET/CT examination, the latter unfortunately not yet being a standard imaging modality in anal cancer. Case 4 was an advanced T4N3M0 tumor with initial involvement of inguinal lymph nodes on both sides with perivulvar lymphovascular invasion on the left side. The infiltrated genital area was included into the PTV. Due to persisting anal tumor and progressive lymph nodes salvage surgery was performed. She later received palliative chemotherapy due to multilocular progression and died a few months later in palliative care.

Case 2 and case 4 therefore show cases with locoregional failure and subsequent systemic progressive disease. In the context of systemic progressive disease the issue of local failure is sometimes of minor prognostic importance, the consequences for the patient (e.g. painful local complications) may nevertheless be severe. However, it has also to be kept in mind that, as recently shown by Mai et al., as well as in other recent publications, local relapse is not salvaged any more at recently published rates and thus dramatically influences overall survival [9], which is supported by our cases. Possibly, uncontrolled local disease increases the risk for systemic spread.

## Conclusions

In conclusion, while IMRT in anal cancer is feasible and improves high-dose-conformality, our case reports show that in both early and advanced anal tumors there is a risk of genital/perigenital spread at time of first diagnosis, increasing the risk for marginal misses if target volumes are too small. Genital spread can occur in form of direct infiltration (which may not only occur in high T-stage) or perigenital lymphovascular invasion (for which a hypothesis suggested by this report is that it may be associated with inguinal N+ disease). Until detailed pattern-of-relapse analyses of controlled IMRT-series such as RTOG-0529 are available, PET/CT and possibly MRI should be considered as a staging tool, genital sparing should be applied with caution, and meeting genital dose constraints should not be prioritized above CTV coverage, especially (but not only) in stage T3/4 and inguinal N+ disease.

## Consent

All procedures followed were in accordance with the ethical standards; an approval by an ethics committee was not applicable. All patients have consented with their regular informed consent to anonymous scientific analysis of their data.

## Abbreviations

3D-CRT: 3D conformal radiotherapy
5FU: 5-fluorouracil
AGITG: Australasian Gastrointestinal Trials Group
CTV: clinical target volume
Gy: gray
HPV: human papillomavirus
IMRT: intensity modulated radiotherapy
MMC: mitomycin C
MRI: magnetic resonance imaging
NCCN: National Comprehensive Cancer Network
OAR: organs at risk
PET/CT: positron emission tomography/computed tomography
PTV: planning target volume
RTOG: Radiation Therapy Oncology Group
SCC: squamous cell carcinoma
